# CRNDE: A Long Non-Coding RNA Involved in CanceR, Neurobiology, and DEvelopment

**DOI:** 10.3389/fgene.2012.00270

**Published:** 2012-11-29

**Authors:** Blake C. Ellis, Peter L. Molloy, Lloyd D. Graham

**Affiliations:** ^1^CSIRO Animal, Food and Health Sciences, Preventative Health Flagship, Commonwealth Scientific and Industrial Research OrganisationSydney, NSW, Australia

**Keywords:** CRNDE, lncRNA, IRX5, cancer, neurogenesis, 4933436C20Rik, multipotency, glioma

## Abstract

*CRNDE* is the gene symbol for *Colorectal Neoplasia Differentially Expressed (non-protein-coding)*, a long non-coding RNA (lncRNA) gene that expresses multiple splice variants and displays a very tissue-specific pattern of expression. CRNDE was initially identified as a lncRNA whose expression is highly elevated in colorectal cancer, but it is also upregulated in many other solid tumors and in leukemias. Indeed, CRNDE is the most upregulated lncRNA in gliomas and here, as in other cancers, it is associated with a “stemness” signature. CRNDE is expressed in specific regions within the human and mouse brain; the mouse ortholog is high in induced pluripotent stem cells and increases further during neuronal differentiation. We suggest that CRNDE is a multifunctional lncRNA whose different splice forms provide specific functional scaffolds for regulatory complexes, such as the polycomb repressive complex 2 (PRC2) and CoREST chromatin-modifying complexes, which CRNDE helps pilot to target genes.

## Introduction

### Gene locus and transcription

**Figure 1:**
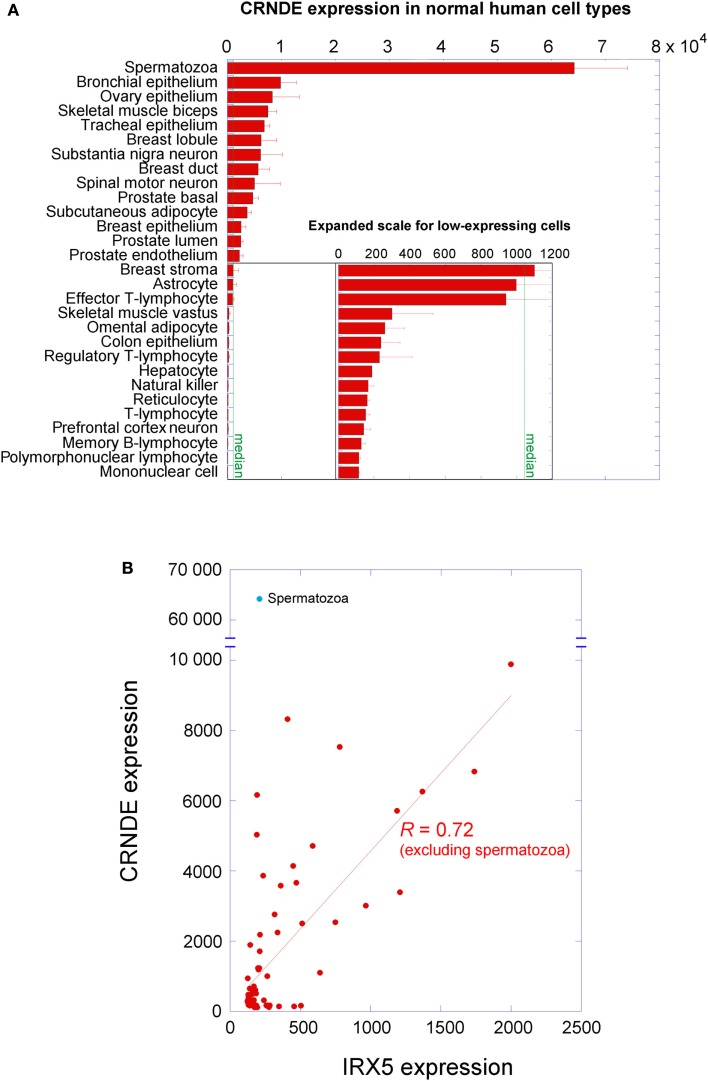
**Expression of CRNDE in normal adult human cell types**. **(A)** Expression levels were measured on Affymetrix HG-U133 arrays; data from the Body Atlas, Cell Types, at http://www.nextbio.com. The median expression across all 67 cell types in the database is shown by the green line. All of the blood cells listed come from peripheral blood. **(B)** CRNDE expression levels plotted against IRX5 expression levels for each cell type in NextBio. An unreplicated experiment on dental odontoblasts with anomalous outcomes for both genes has been omitted. When spermatozoa are excluded, the two genes show correlated expression (*R* = 0.72 in linear regression by KaleidaGraph v3.6, Synergy Software).

**Figure 2:**
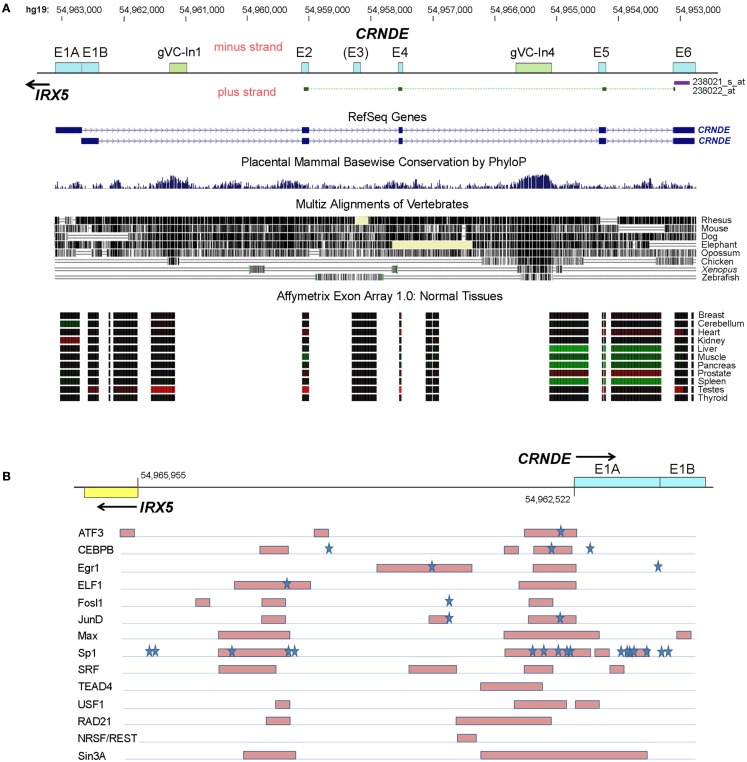
**Human *CRNDE* gene locus**. **(A)** Nucleotide numbering (top) is for chromosome 16, hg19. Below that is the *CRNDE* locus, oriented 5′ to 3′ (left to right). Exons are shown as boxes with cyan fill; the most highly conserved regions of the locus, which are intronic, are shown as boxes with olive-green fill. The target regions of the two HG-U133 probesets are shown immediately below the locus baseline. The two RefSeq genes are shown; fully spliced AceView transcripts (not shown) typically commence with just a short segment from the 3′ end of one of the two alternative first exons, E1A and E1B. Evolutionary conservation within the locus is shown for placental mammals (PhyloP) and for vertebrates in general (Multiz alignments). The bottom of this panel shows the expression of *CRNDE* exons and some intronic regions in normal human tissues, obtained using the Affymetrix Exon Array 1.0; the data for each probeset are color-coded (red, expression higher than the median value across all tissues, green, expression lower than the median). Source of data below locus diagram: UCSC Genome Browser (http://www.genome.ucsc.edu), minus-strand display. **(B)** First exons of *CRNDE* and *IRX5* and the intervening promoter-containing region (∼2 kb) between their transcription start sites. Transcription factor (TF) binding was identified from “Transcription factor Binding Sites by ChIP-seq from ENCODE/HAIB” tracks on UCSC Genome Browser. Regions of strongest binding in HCT116 cells by TFs (listed on the left) are shown by pink shaded boxes. Blue stars indicate the locations of predicted strong TF DNA binding motifs, from the TRANSFAC database (Wingender, 2008).

*CRNDE* is the gene symbol for *Colorectal Neoplasia* *Differentially Expressed (non-protein-coding)*, a human gene locus whose expression was identified by our laboratory as upregulated in colorectal adenomas and carcinomas (Graham et al., 2011). *CRNDE* is transcribed from chromosome 16 on the strand opposite to the adjacent IRX5 gene, with which it may share a bidirectional promoter. The locus produces transcripts with low coding potential, which has resulted in its classification as a long non-coding RNA (lncRNA; Cabili et al., 2011). CRNDE displays tissue-specific and temporal expression patterns, in that it has little to no expression in many normal adult tissues, such as colorectal mucosa, white blood cells, and liver, while tissues with high CRNDE expression include testis, breast, skin, parotid gland, and bronchial epithelium (data from NextBio^1^, BioGPS^2^, and the UCSC Genome Browser^3^). Expression levels in a selection of adult cell types are shown in Figure 1A and expression of individual exons in selected tissues at the bottom of Figure 2. CRNDE expression can vary considerably within a tissue category, such as the different types of adipose tissue or skeletal muscle (Figure 1A), as well as within a particular organ. The brain is an example of a complex organ that shows differential CRNDE expression within its various compartments and structures; it will be discussed in detail below.

The *CRNDE* locus spans ∼10 kb and comprises five core exons, with an additional exon (E3) that is infrequently present in transcripts (Figure 2A). Complex alternative splicing results in transcripts that contain different combinations of exons, as well as varying amounts of retained intronic sequence (Graham et al., 2011); many additional permutations have subsequently been identified in our laboratory. A recent ENCODE survey found that the efficiency of co-transcriptional splicing decreases progressively with proximity to the polyadenylation site and noted that lncRNAs are especially prone to incomplete splicing (Tilgner et al., 2012). Consistent with this, the retention of CRNDE intronic segments seems mainly to involve the downstream introns, while RNA-Seq profiling of the CRNDE locus (UCSC Genome Browser, hg19 ENCODE RNA-Seq tracks) indicates that its upstream introns – especially the first one – are removed efficiently.

Subcellular fractionation within HCT116 and HT29 CRNDE-expressing colorectal cancer (CRC) cell lines revealed that transcripts that have retained intronic sequence are highly enriched in the nucleus (Ellis et al., 2012); typically, greater intronic retention results in higher nuclear enrichment. A nuclear localization is in accordance with general findings for lncRNAs (Derrien et al., 2012) and is consistent with their putative roles in transcriptional regulation. Unexpectedly, we found that the fully spliced CRNDE transcripts that contain only exon sequences are enriched in the cytoplasm, a distribution typical of exonic protein-coding transcripts. Evidence is emerging that some translation of CRNDE’s short open reading frames (sORFs) is possible (see below).

A survey within the ENCODE project showed that the expression of almost 3% of lncRNAs shows high positive correlation with that of a neighboring mRNA (Derrien et al., 2012). The *CRNDE* promoter resides in the same CpG island as that of the adjacent *IRX5* gene, and the presumed promoter regions exhibit coordinated methylation (Graham et al., 2011). We had also noticed that some expression profile comparisons showed strongly concordant differential expression of CRNDE and IRX5; for example, CRNDE and IRX5 ranked first and third in the list of genes differentially expressed in a comparison of subcutaneous with visceral adipose tissue (VAT) depots (72- and 54-fold upregulation, respectively; Wolfs et al., 2010). A survey of NextBio data for normal human cell types showed that, in general, the expression of CRNDE does indeed correlate positively (*R* = 0.72) with that of IRX5 (Figure 1B). The striking exception to this relationship occurs in spermatozoa, where CRNDE expression is very high but IRX5 expression is very low; this may reflect a special role for CRNDE in gametogenesis (discussed further below).

Recent data published as part of the ENCODE project have included the mapping of genomic regions bound by specific transcription factors (TFs) following immunoprecipitation of fragmented chromatin (TF-IP). This includes data on the CRNDE-expressing CRC cell line HCT116 (data from Richard Myers’ laboratory; ENCODE Project Consortium et al., 2012). A representation of regions and TFs showing the highest levels of binding across the *CRNDE*-*IRX5* promoter regions is shown in Figure 2B. Strong binding of TFs is particularly evident in two regions, one encompassing the *CRNDE* exon E1A and immediately upstream of it, and a second region upstream of the *IRX5* transcription start site. As well as identifying sites of direct DNA binding by TFs, TF-IP reveals sites where TFs do not bind directly to DNA but bind via complexes with other TFs or chromatin components. In a number of the examples shown in Figure 2B, direct binding can be inferred from the presence of potential TF binding sequences within the captured regions (e.g., ATF, CEBPB). Frequently, TF-IP demonstrates binding of a specific TF to both the *CRNDE* and *IRX5* promoter regions (e.g., ATF3, CEBPB, Max, and Sp1), supporting the observation that the expression of both genes is under a level of co-ordinate control (Figure 1B). While the survey of TF binding to the *CRNDE*-*IRX5* promoter regions is necessarily limited, it does indicate that their expression is subject to upstream signaling pathways. ATF3 binding suggests responsiveness to CREB/ATF signaling, while Fosl and JunD and SRF binding are associated with response to serum components (via the MAP kinase pathway, for example) and with cell cycle, growth, and differentiation. Binding of the Myc partner protein, Max, also implicates *CRNDE* as a target of the Myc regulatory pathway. Of possible significance is the binding to the *CRNDE* promoter of Sin3A and REST, which form part of the CoREST complex; CRNDE RNA has been shown to associate with CoREST (Khalil et al., 2009), perhaps indicating its involvement in a *cis*-acting regulatory pathway.

Loci orthologous to *CRNDE* are identifiable in other vertebrates. Curiously, the sequences in the *CRNDE* locus that are most evolutionarily conserved fall within the introns rather than the exons, and we have identified two genomic Vertebrate Conserved (“gVC”) regions, one in Intron 1 (“gVC-In1”) and the other in Intron 4 (“gVC-In4”). gVC-In1 and gVC-In4 are highly conserved among vertebrates as far as the chicken, with gVC-In4 conservation extending to *Xenopus* and zebrafish (Figure 2). The gVC-In4 core extends over roughly 200–300 nucleotides and contains two conserved secondary structures (EvoFold 72 and 76)^4^. Bioinformatic analysis and our experimental data indicate that the gVC-In4 segment can be included within longer exon-containing transcripts, but it may also be expressed independently. It therefore seems to behave like both a transcribed ultraconserved region (“T-UCR”; Calin et al., 2007) and an additional or alternative exon. Considering that the *CRNDE* locus as a whole is poorly conserved across species, the role and function of gVC-In4, a predicted regulatory element, are of particular interest.

Transcripts from genes homologous to human *CRNDE* are found in many other mammals (e.g., chimpanzee, baboon, rhesus macaque, cow, rat, mouse, and dog). In such transcripts, there is a general tendency toward omission of E2 and E5 and the appending of additional exons downstream of E6. This is true of the mouse ortholog of *CRNDE* (gene symbol: 4933436C20Rik), which will feature repeatedly in our review. For convenience we will refer to it as “mouse *CRNDE*.” Clark et al. (2012) found that a transcript from mouse *CRNDE* (there called “linc1399,” Table A1 in Appendix) is notable for being very stable, with a half-life of 13.6 h; only 51 of the 823 lncRNAs surveyed in their study had half-lives >12 h. There is currently no information on the stability of human CRNDE transcripts. While RNA longevity is consistent with a function as a cellular housekeeper (Clark et al., 2012), we find that CRNDE does not appear to behave as such.

### CRNDE as a lncRNA

As a newly identified gene, few publications address CRNDE’s regulation and function directly; however, it features repeatedly in the supplementary data of journal publications and in the bioinformatics data in public repositories. Several lines of evidence suggest that *CRNDE* exerts its effects primarily through its RNA transcripts. First, bioinformatic analyses identified CRNDE (under its various aliases, Table A1 in Appendix) as a putative non-coding RNA. Its coding potential score is −1.85; the scores for other lncRNA candidates range from −36 to +757 (Derrien et al., 2012). Second, the study by Khalil et al. (2009) demonstrated direct interaction between CRNDE transcripts and components of the polycomb repressive complex 2 (PRC2) and CoREST complexes. This study also found that there was an overlap in the lists of genes affected by short interfering RNA (siRNA)-mediated knockdown of CRNDE and PRC2, potentially implicating CRNDE in the epigenetic remodeling of chromatin, and particularly in the downregulation of gene expression via targeted histone methylation or demethylation by PRC2 or CoREST complexes, respectively (Khalil et al., 2009). Third, comparative genomics reveals that the features required for the translation of the most obvious of the human sORFs (Graham et al., 2011), such as start/stop codons, splice donors/acceptors, and reading frame integrity, are poorly conserved in mammals more distantly related to us than chimpanzee. Fourth, we have shown that the phenotypic effects associated with the knock-in of the sORFs (Graham et al., 2011) into CRC cell lines is unaffected by the deletion of their start codons (Ellis et al., 2012), which indicates that the effects are mediated by the transcripts rather than by any polypeptides that they may encode. We have also been unable to achieve *in vitro* expression of CRNDE polypeptides.

In the ENCODE project, a large-scale mass spectrometry survey found that human lncRNAs are rarely translated (Bánfai et al., 2012). In contrast, a recent study by Ingolia et al. (2011) observed that the majority of mouse lncRNAs contain regions of high translation, with mouse CRNDE transcripts showing a low but non-zero ribosome density. In addition, a specialized mass spectrometry survey by Granados et al. (2012) identified the CRNDE peptide LQFIMELLY as being presented by the major histocompatibility complex 1 (MHC1) of Epstein–Barr virus-immortalized B-lymphocytes. Granados et al. (2012) describe the immunopeptidome as arising from defective ribosomal products which do not fold correctly. It is possible that the translation of CRNDE sORFs is adventitious and that the polypeptides are unable to form stable folds, in which case all of the translated material would be available for presentation by the MHC1 complex. Altogether, despite an awareness that fully spliced CRNDE transcripts accumulate in the cytoplasm (Ellis et al., 2012) and that some translation of their sORFs can occur (Granados et al., 2012), there is currently no evidence that CRNDE polypeptides serve as important mediators of the gene’s function.

## CRNDE Expression in Cancers

### Colorectal cancer

We first identified *CRNDE* as a previously uncharacterized gene locus (hCG_1815491, Table A1 in Appendix) that was differentially expressed in colorectal neoplasia relative to normal colorectal tissue, and in particular was among the most differentially expressed genes in pre-cancerous adenomas (Graham et al., 2011). Elevated CRNDE expression was also identified in independent datasets of adenoma and CRC gene expression (Hong et al., 2007; Sabates-Bellver et al., 2007; Kaneda et al., 2010; Nagaraj and Reverter, 2011). Subsequently, our preliminary data from knock-in of regions from CRNDE transcripts and from siRNA-mediated knockdown studies of gVC-In4-containing transcripts in CRC cells have indicated that CRNDE transcripts can promote growth and suppress apoptosis (Ellis et al., 2012). This supports a role for CRNDE as a mediator of oncogenesis.

### Other cancers

Whereas we discovered *CRNDE* as a gene upregulated in colorectal neoplasia, it is evident that CRNDE expression is increased in a variety of other neoplastic diseases, especially cancers of the blood and brain. Table 1 highlights a number of cancer types in which CRNDE expression is significantly altered. These include both solid tumors and leukemias. Interestingly, elevated CRNDE expression is typically found in cancers that develop from a cell type or organ in which it is not normally expressed (e.g., liver, colon, kidney, leukocytes), suggesting that its presence gives the tumor cell an advantage not normally required by that cell type. In leukemias and some solid tumors, it is evident that altered CRNDE expression is characteristic of different subclasses and may be associated with different clinical outcomes. While moderately upregulated in most acute myeloid leukemias (AML), CRNDE expression is particularly elevated in FAB Type M2 AML (Le Dieu et al., 2009) and in promyelocytic Type M3 AML (Payton et al., 2009). CRNDE transcripts are among those whose expression is most elevated (21-fold relative to non-cancerous tissue) in hepatocellular carcinoma (HCC), with a greater increase (35-fold) in the HCC subset correlating with high JNK1 activity. While CRNDE expression is mildly elevated in prostate cancer (Lapointe et al., 2004; Pressinotti et al., 2009), the upregulation is associated with a subgroup of prostate cancers with poor prognosis, and about one-third of metastases express CRNDE at elevated levels (Taylor et al., 2010; Markert et al., 2011).

**Table 1:** **CRNDE expression in different cancers**.

Cancer		Fold change	Reference
Colorectal cancer	Adenomas	4.6, 41, 14.2	Graham et al. (2011), Nagaraj and Reverter (2011), Sabates-Bellver et al. (2007)
	Cancer	6.1, 16.1	Graham et al. (2011), Nagaraj and Reverter (2011)
Hepatocellular carcinoma	Upregulated compared to matched normal liver	21	Chang et al. (2009)
Kidney	Clear cell carcinoma vs. matched normal	2.3–5.95	Cifola et al. (2008), Gumz et al. (2007), Ooi et al. (2011), Kort et al. (2008)
	Type 1 papillary renal tumor	3.4, 4.6	Ooi et al. (2011), Kort et al. (2008)
	Chromophobe renal tumor	−7.9, −4.2, −6.4	Ooi et al. (2011), Kort et al. (2008), Yusenko et al. (2009)
	Oncocytoma	−5.5, −5.6	Kort et al. (2008), Yusenko et al. (2009)
Adrenal	Adrenocortical carcinoma vs. normal adrenal cortex	5.7	Giordano et al. (2009)
Pancreatic cancer	Pancreatic cancer vs. matched normal tissue	2.9, 4.2, 5.4	Ishikawa et al. (2005), Pei et al. (2009), Donahue et al. (2012)
Prostate cancer	Mildly upregulated	1.3, 1.6	Pressinotti et al. (2009), Lapointe et al. (2004)
	Associated with progression		Markert et al. (2011), Taylor et al. (2010)
Ovarian cancer	Serous ovarian tumors or tumor epithelial cells compared to normal	−10 to −54	Shahab et al. (2011), King et al. (2011)
AML	*CRNDE* is one of 731 genes whose expression forms an AML signature	5.5–5.8	Rager and Fry (2012)
	CD4(+) or CD8(+) T-cells of FAB type M2 AML patients compared with normal cells	44–55	Le Dieu et al. (2009)
Acute promyelocytic leukemia	AML M3 subtype. Bone marrow compared with healthy marrow [CD34(+) cells, promyelocytes or neutrophils]	10.2–89	Payton et al. (2009)
Multiple myeloma (MM)	CRNDE probesets are the fourth and tenth most upregulated in one novel cluster, characterized by overexpression of cancer/testis antigens without overexpression of proliferation genes	4.2–4.8	Broyl et al. (2010)
	Part of a “spike” gene set associated with specific translocation in MM		Kassambara et al. (2012)
T-cell leukemia	CD4(+) T-cells from adult T-cell leukemia, compared to normal	32	Choi et al. (2007)
	Bone marrow of T-cell acute lymphoblastic leukemia relative to normal	15	Kohlmann et al. (2008)
Cutaneous anaplastic large cell lymphoma (ALCL)	ALCL relative to range of T-cell controls (tonsil and peripheral blood)	55–62	Eckerle et al. (2009)
Gliomas	Average across different glioma types and grades relative to non-tumoral brain tissue controls	14–32	Zhang et al. (2012)

While CRNDE expression is most often elevated in cancers, in some cases it is lower than in normal control tissue. For example, in contrast to clear cell carcinomas and Type I papillary tumors of the kidney, chromophobe tumors and oncocytomas show significantly downregulated expression of CRNDE (Table 1). For serous ovarian carcinoma, significantly decreased expression (10- to 54-fold) relative to normal ovarian epithelium has been observed (King et al., 2011; Shahab et al., 2011), and levels differ markedly between different sub-types of ovarian cancer (Mateescu et al., 2011; GSE26193)^5^. Some tumors with low CRNDE may simply represent cases where the cell type of origin expresses little CRNDE and it does not increase with neoplasia.

While de-regulated CRNDE expression – and particularly elevated expression – is common to many cancers, we have little understanding of whether its upregulation confers an oncogenic phenotype, as it potentially does in CRC. For many of the published studies from which we have drawn data, the detection of expression is based on just two probesets in the Affymetrix HG-U133 arrays. Of these, the first (238021_s_at) targets the 3′-terminal exon, E6, and thus detects almost all of the canonical AceView-listed CRNDE transcripts, almost all of which include E6 (aAug10-lAug10)^6^. The second probeset (238022_at) detects fully spliced transcripts across exons E2, E4, E5, and E6, and therefore detects only a specific subset of CRNDE exonic transcripts. There are often large differences in the extent of differential expression displayed by each probe: between normal and cancer samples, the E6 probeset signal typically differs more. As mentioned above, we know of many unspliced, nuclear-enriched CRNDE transcripts which would only be detected by the E6 probeset. If this category is more elevated in cancer, it would suggest a link between the CRNDE transcripts that interact with chromatin-modifying complexes in the nucleus and those that are most correlated with tumorigenesis. Given the complex repertoire of splice variants of CRNDE, it will be important to identify/characterize in more detail the transcript profile in different cancer types. Unfortunately, probes for CRNDE transcripts were not included in the GENCODE v3c lncRNA microarray (Agilent) designed by the ENCODE consortium (Derrien et al., 2012).

## CRNDE Expression in Development

### Multipotency

CRNDE expression appears highest in the early stages of human development and progressively decreases thereafter, with detected expression ranking as follows (transcripts per million in parentheses): embryoid body (42) > fetus (25) > juvenile (17) > adult (6) (UniGene EST database)^7^. The tentative link between CRNDE expression and less specialized cell types has recently received some very direct support, with mouse CRNDE being implicated as one of several lncRNAs required for the maintenance of pluripotency in mouse embryonic stem cells (moESC), and thus potentially involved in tumorigenesis (Guttman et al., 2011). Specifically, at the locus for mouse *CRNDE* (“linc 1399,” Table A1 in Appendix), the promoter was bound by select pluripotency-related TFs, including *c*-Myc and *n*-Myc, while knockdown of CRNDE was associated with decreased levels of a different suite of critical pluripotency markers: Sox2, Klf4, Nanog, and Oct4.

Guil et al. (2012) have shown that intronic segments from several lncRNAs bind to PRC2 and recruit the complex to their genes of origin, which then undergo epigenetic downregulation. Although a direct association between CRNDE transcripts and PRC2 has not been detected in moESC (Zhao et al., 2010), both PRC1 and PRC2 bind to and down-regulate transcription from the *CRNDE* promoter in such cells (Boyer et al., 2006; Endoh et al., 2008; Pasini et al., 2008). The hESC cell line H1 also has relatively low levels of CRNDE expression (Derrien et al., 2012). A negative feedback loop directed by CRNDE intronic sequences could account for these observations.

Consistent with a function in cell pluripotency and/or an undifferentiated state, as suggested in the study by Guttman et al. (2011), T-cells exhibit a progressive decrease in CRNDE levels during progression from the intrathymic T progenitor stage to naïve T-cells; specifically, they drop after the transition from CD4(+)CD8(+) double-positive to CD4(+)CD8(−) single-positive T-cells (Lee et al., 2004; GDS786). The dramatic increase in CRNDE (44- to 55-fold) in both CD4(+) and CD8(+) cells of AML patients (Le Dieu et al., 2009) suggests that loss of CRNDE expression may correlate with the transition to a single-positive stage rather than with selection of CD4(+) over CD8(+). Sustained CRNDE expression during T-cell maturation may impede the cell’s proper differentiation into single-positive cells, perhaps forcing it to remain as a multipotent progenitor cell. The extent to which CRNDE is elevated in blood cancers is quite remarkable, and is consistent with an activation of stem or progenitor cell expression programs in cancers (Table 1). This is also evident in prostate cancer, where elevated CRNDE expression forms part of a stem cell-like gene signature associated with a subset of more aggressive cancers (Markert et al., 2011).

The complexity of developmental regulation is underscored by a counter-example in which CRNDE expression is persistently downregulated following telomerase-induced immortalization of human urothelial cells (Chapman et al., 2008). One might have expected CRNDE to rise in these cells, which are less able to differentiate, but instead its expression is attenuated. A number of genes involved in differentiation also showed decreased expression, raising the possibility that – in specific cellular contexts – CRNDE transcripts might also participate in differentiation processes.

### Differentiation

In addition to an apparent role for CRNDE in maintenance of cell pluripotency, several studies implicate CRNDE as important in cellular differentiation. During differentiation, CRNDE expression may be silenced or – depending upon the specific lineage – may persist or increase, suggesting a potential role in lineage determination. For example, Lin et al. (2011) observed CRNDE to be one of the most highly expressed lncRNAs in human induced pluripotent stem cells (iPSCs), but even more importantly, to be one of the genes most highly induced during their subsequent differentiation into neurons (Lin et al., 2011). This work will be considered further below. Consistent with these findings, CRNDE expression is also elevated (relative to human embryonic stem cells, hESC) following induction into multipotent mesodermal progenitor CD326(-)/CD56(+) cells using activin A, bone morphogenetic protein 4, vascular endothelial growth factor, and fibroblast growth factor 2 (Evseenko et al., 2010; E-GEOD-21668)^8^. Other progenitor cell types also show elevated CRNDE expression with differentiation. CD34+ hematopoietic progenitor cells that were induced to differentiate using erythropoietin, interleukin-3, and stem cell factor showed a 6.1-fold increase in CRNDE levels from days 1 to 11 (Keller et al., 2006; GDS2431)^9^. Airway primary cells differentiating into polarized, pseudostratified epithelia demonstrated a small but progressive increase (∼2-fold) in CRNDE expression over the 28-day experiment (Ross et al., 2007; GDS2615)^10^. Preadipocytes from omental adipose tissue, but not those from subcutaneous adipose tissue (SAT), show very low expression of CRNDE, in a similar pattern to that of *HOX* and *IRX* gene family members (including the *IRX5* gene adjacent to *CRNDE*), but CRNDE expression is markedly upregulated in differentiated omental adipocytes (∼10-fold; Tchkonia et al., 2007). Altogether, the evidence suggests that high CRNDE expression is associated with pluripotency, but that further increases may accompany the process of differentiation into specific lineages. It is therefore tempting to speculate that certain CRNDE transcripts maintain pluripotency, while others specify cell lineage and orchestrate progression of the cell toward that destination.

Beyond the findings of Tchkonia et al. (2007), there is an apparent difference in overall CRNDE expression between VAT and SAT. For example, Wolfs et al. (2010) identified *CRNDE* as the top differentially expressed gene in SAT relative to omental VAT, with a 72-fold increase in the former. It was observed in both adipose tissue studies that genes whose expression is favored in subcutaneous adipocytes, including members of the *HOX* and *IRX* families, tend to correlate with ectoderm development pathways, while genes expressed in VAT represent those of mesoderm development. The higher expression of CRNDE in SAT relative to omental VAT is consistent with a general trend we have observed for substantial CRNDE expression in normal adult tissues of ectodermal origin, e.g., skin, breast, central nervous system (discussed below).

### Gametogenesis

In general, lncRNAs show relatively high expression in testicular tissue (Derrien et al., 2012), and CRNDE expression itself is highest in the testes in both human and mouse (BioGPS, NextBio, UCSC Genome Browser). CRNDE levels are 5.5-fold greater in human testicular samples that contain spermatogonia compared to those that contain no germ cells (von Kopylow et al., 2010). A couple of independent studies reveal a rise in CRNDE expression during the progression from murine spermatogonia (types A and B), which are diploid, to secondary spermatocytes and spermatids, which are haploid (and correspond to the meiotic and postmeiotic stages, respectively; Namekawa et al., 2006; Chalmel et al., 2007). A third study suggests that CRNDE expression decreases during the progression from secondary spermatocytes to early (i.e., round) spermatids (E-GEOD-21749). Altogether, it might be concluded that CRNDE expression rises in the approach to meiosis, during which it peaks, and remains high relative to other tissues at the completion of spermatogenesis (spermatozoa, Figure 1A). In view of its association with cancer, the high expression of CRNDE in meiotic/postmeiotic sperm cells is noteworthy considering the existence of a “cancer/testis antigen” (CTA) category of genes, a correlation that implies connections between spermatogenesis and somatic tumorigenesis (Fratta et al., 2011).

## CRNDE in Neurobiology

### Expression in brain

**Figure 3:**
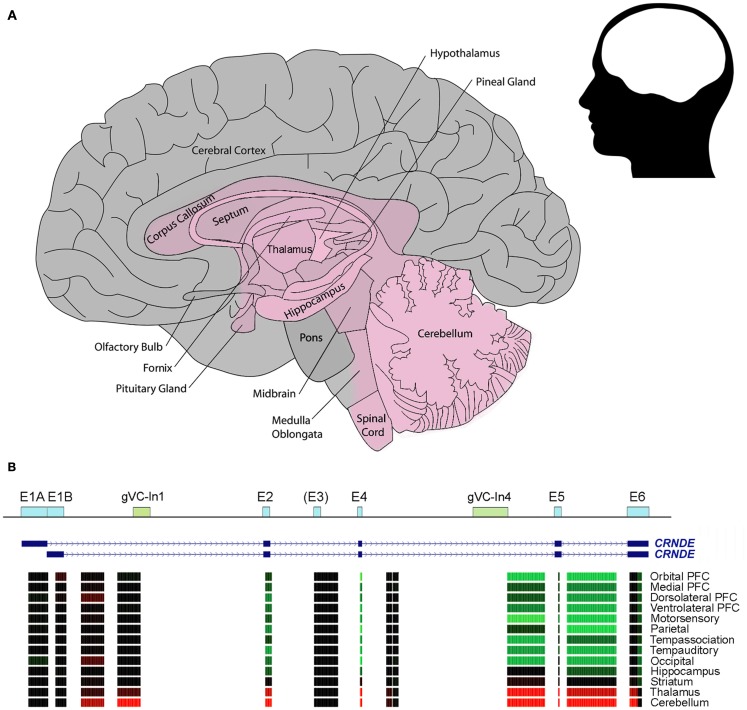
**CRNDE expression in the human brain**. **(A)** Schematic showing the region where CRNDE is expressed in the adult human brain. CRNDE expression (shaded pink) is concentrated in the deep central structures (especially the basal ganglia and adjacent structures), cerebellum, posterior brain stem, and spinal cord. In the CRNDE-expressing region, no attempt has been made to display differences in expression levels between or within structures. **(B)** Affymetrix Human Exon 1.0 ST microarray expression data from the late mid-fetal human brain, generated by the Sestan Lab at Yale University; these are grouped by the median for each brain region and color-coded as in Figure 2A. The first nine regions are from the neocortical region of the cerebral cortex; PFC, pre-frontal cortex. Data sources: http://www.molecularbrain.org, the Allen Human Brain Atlas (Brain Explorer 2), and Sestan Human Brain Atlas (UCSC Genome Browser).

We have seen above that CRNDE is expressed during neural differentiation from human iPSCs (Lin et al., 2011). Although this study reported that CRNDE was detectable in fetal but not in adult normal brain tissue, we now know that CRNDE displays highly specific spatial expression within the substructures of the mature human, rat, and mouse brain; in this case, the adult brain samples came from the pre-frontal cortex, where CRNDE expression is typically low or absent. In the human brain, expression is predominant in the deep central structures, especially the basal ganglia and adjacent structures (nucleus accumbens, globus pallidus, head of caudate nucleus, putamen, thalamus, and corpus callosum), as well as the cerebellum, posterior brain stem (midbrain, pons, medulla oblongata), and spinal cord (Figure 3A). Like CRNDE, some lncRNAs involved in neurogenesis are expressed in fetal brain and in the mature midbrain (substantia nigra) and cerebellum (Ng et al., 2011).

The relative expression levels of CRNDE segments in some types of human brain cells are shown in Figure 3B. Notably, exon array data show that there is significant expression of sequences within Intron 1; one of these probesets overlaps the conserved gVC-In1 region. This region is not well expressed in other tissues except for the testes (Figure 2A). From a comparison of expression data for human, rat, and mouse brains, which have been normalized to β_2_-microglobulin, it appears that there may be higher expression of CRNDE in rodent than in human brains; the maximum expression values detected for the three species were 5, 45, and 37, respectively (Table 2). The highest level recorded for mouse brain is in the olfactory bulb (Table 2), the end of a tract protruding from the CRNDE-expressing structures of the brain (Figure 3A), which may be particularly important to rodents. Significant expression has also been reported for some of the frontal cortex of rodent brains, and for human frontal lobe gyri close to the deep central structures and the olfactory tract (i.e., parts of the orbital, paraterminal, and subcallosal gyri; not shown in Figure 3A).

**Table 2:** **Relative expression levels of CRNDE in brains of humans and rodents^a^**.

	MolecularBrain.org^b^			GeneNetwork.org^c^	
	Human	Rat^d^	Mouse	Mouse	
				First exon	Last exon
Striatum	–	8	31	10.6	9.7
→ Caudate nucleus	0	–	–	–	–
→ Nucleus accumbens	–	45	–		–
Thalamus	4	–	–	–	–
Hypothalamus	4	14	4	–	–
Hippocampus	3	11	1	10.4	9.9
Olfactory bulb	–	–	37	–	–
Amygdala	1	3	0	–	–
Pineal	–	24	–	–	–
Cerebellum	5	27	0	–	–
Pituitary	1	1	1	–	–
Spinal cord	5	9	2	–	–
Frontal cortex	0	7	16	–	–

*^a^Expression microarray data from public databases. “–” denotes no data*.

*^b^Values from http://www.molecularbrain.org, where expression is normalized to β_2_-microglobulin*.

*^c^Values from http://www.genenetwork.org for RMA-normalized Affymetrix exon array probesets 5051610 and 5117873, which target the first and last exons of the RefSeq gene, respectively; these are the two highest-expressing regions of the locus. The lowest values (for an intronic probeset) were 5.7–5.8. Accession number for striatum data is GN163, that for hippocampus is GN206*.

*^d^The rat gene orthologous to CRNDE is RGD1565975*.

### Expression in brain cancers

CRNDE expression is elevated in gliomas. This is particularly true for glioblastomas (highest), astroblastomas, and astrocytomas, whereas oligodendrogliomas and oligoastrocytomas show less dramatic differences (and in some cases none) from normal tissue (Sun et al., 2006; Turkheimer et al., 2006; Murat et al., 2008; Pollard et al., 2009; Paugh et al., 2010). In all tumor types, there tends to be a positive correlation with grade and recurrence. Interestingly, a recent study used publicly available Affymetrix HG-U133 array data to profile lncRNA expression across different types and grades of human gliomas. Of the 129 lncRNAs identified as differentially expressed in gliomas, the results consistently and convincingly identified CRNDE (both probesets) as the most highly upregulated lncRNA (32-fold; Zhang et al., 2012). This was the case for low and high grade astrocytomas (15- and 36-fold, respectively) and oligodendrogliomas (8- and 29-fold, respectively), and CRNDE levels correlated positively with tumor grade in both types. Along with CRNDE, HOTAIRM1 was also one of the most upregulated lncRNAs in astrocytomas. Furthermore, CRNDE is highly expressed in glioblastoma cell lines (LN229, LN215, LN319, LN018, BS149) relative to normal brain (16- to 23-fold up) as well as in primary glioblastomas (3.5- to 6.7-fold up; Grzmil et al., 2011). Gliomas are unique in the degree of neovascularization they exhibit, which allows for their invasion into surrounding brain tissue, making surgical removal difficult. They also exhibit a high degree of pluripotency, similar to that of stem cells (reviewed by Watkins and Sontheimer, 2012), once again correlating high CRNDE expression with stemness.

Another study revealed a positive correlation between CRNDE levels and epidermal growth factor receptor (*EGFR*) gene amplification, associated with EGFR overexpression, in high grade oligodendrogliomas, a type of glioma in which CRNDE is not always upregulated (Ducray et al., 2008). This study compared tumor tissues with 1p19q codeletion vs. *EGFR* amplification, and found that CRNDE expression was 37-fold higher in the latter and at levels comparable to those seen in CRNDE-expressing gliomas (i.e., glioblastomas, astroblastomas, and astrocytomas); expression in tumors with the codeletion was very low. The authors observed parallels between genes expressed in 1p19q codeletion gliomas and those in normal brain tissue. On the other hand, they note that transcriptomic signatures induced by EGFR overexpression mimic those of glioblastoma stem cells. In support of this, EGFR expression inversely relates to responsiveness of the tumor to differentiation stimulants (Hoi Sang et al., 1995). *EGFR* amplification therefore is associated with maintenance of or reversion to a multipotent state. The intriguing relationship between CRNDE and EGFR overexpression, and potentially cell potency/differentiation, may extend to and have ramifications in other cancers. CRNDE’s participation in various signaling pathways is currently being investigated in our laboratory.

### Involvement in neural development

Brain development and neural differentiation are complex processes that rely upon epigenetic changes. These involve both histone modifications and DNA methylation. DNA methylation is believed to play a role in neural stem cell (NSC) differentiation into neurons or glial cells, such as astrocytes and oligodendrocytes, as well as in glioma formation. Neurogenesis gives rise to NSC, which in early gestation only have the potential to become neurons, but in late gestation gain the potential to become astrocytes and oligodendrocytes (Qian et al., 2000). One example of DNA methylation-induced regulation of neurogenesis involves IL-6 signaling in astrocyte specification of NSC. During gestation, DNA methylation determines whether NSC develop into neurons or astrocytes (Takizawa et al., 2001). In late gestation, IL-6-induced demethylation of STAT3 binding sites within promoters of genes specifying astrocyte differentiation allows for binding by the STAT3 TF, which transactivates genes necessary for astrocyte formation (Takizawa et al., 2001; Hatada et al., 2008). Methylation of these promoters appears to be a key regulatory step in neuronal vs. astrocyte specification as it prevents STAT3 binding even when the appropriate signals for astrocyte differentiation are available (Namihira et al., 2008).

Histone modifications are also important for maintenance of stem cell pluripotency and glioma cell proliferation (Suvà et al., 2009; Orzan et al., 2011). HDAC inhibition has been shown to induce neuronal over oligodendrocyte differentiation by reverting the cell from an oligodendrocyte-specific fate toward neuronal proclivity (reviewed by Namihira et al., 2008; Mercer et al., 2010). As one example, HDAC1 and HDAC2 disrupt β-catenin-TCF7L2 interaction, which is normally triggered by Wnt signaling and linked with antagonism of oligodendrocyte differentiation. HDACs compete with β-catenin for binding to TCF7L2, and cause the transcription of genes that induce oligodendrocyte differentiation (Ye et al., 2009).

Many examples now exist for the association of lncRNAs with chromatin-modifying complexes such as the PRC2 complex, or with specific TFs. From this work, a picture is emerging that lncRNAs are key mediators of epigenetic programming through the targeting of such complexes to specific chromosomal sites. Expression of lncRNAs in the brain is highly specific, both spatially and temporally, and it is proposed that they are key regulators of neural differentiation (Qureshi et al., 2010). This is supported by studies that directly implicate specific lncRNAs (e.g., MALAT1, Evf2) in the promotion of neuronal differentiation and that show the expression of lncRNAs during the glial/neuronal specification stage of development (Bond et al., 2009; Bernard et al., 2010; Mercer et al., 2010).

Similar to the Guttman et al. (2011) study in moESC, Ng et al. (2011) recently performed a global study in hESC to investigate the lncRNAs necessary for pluripotency, as well as those necessary for neuronal differentiation. Neurons induced to differentiate from hESC showed differential expression of nearly one thousand lncRNAs from those represented on their array (which unfortunately did not include probes targeting CRNDE). Targeted knockdown of different lncRNAs was shown to either induce or prevent neuronal differentiation. The authors demonstrated the interaction of these lncRNAs with chromatin-modifying proteins such as SUZ12 (a subunit of PRC2) and REST (a binding partner for CoREST), which may mediate neuronal cell fate specification. The paper demonstrates the likely role of multiple lncRNAs in stem cell maintenance as well as neuronal differentiation, and the potential involvement of PRC2 and CoREST complexes, with which CRNDE RNA has been shown to associate.

Though evidence of a functional role in human neurogenesis is lacking, Lin et al. (2011) gave special mention to CRNDE as one of the highest-expressing intergenic lncRNAs within iPSCs, and one which furthermore exhibited one of the largest elevations during neuronal differentiation. CRNDE expression (identified by RNA deep sequencing and validated by quantitative PCR) increased 4.3-fold over 10 days (the earliest time point measured) following induction of neurogenesis. Similarly, Denis et al. (2011) identified CRNDE as being upregulated in neural progenitor cells (but not in mesenchymal progenitor cells) relative to hESC (2.5-fold up). Lin et al. (2011) suggest that lncRNAs – including CRNDE – are involved in the control of neural specification, which raises the question of whether the absence of CRNDE during differentiation might allow development into non-neuronal lineages. Either way, we have shown above (Figure 1B) that expression from the *CRNDE* locus is coordinated with that of *IRX5*, which belongs to a gene family involved in brain patterning and neurogenesis (Cohen et al., 2000; Anselme et al., 2007). Both CRNDE and IRX5 are highly expressed in the thalamus and cerebellum relative to other brain regions (Sestan Human Brain Atlas).

Evidence is also emerging that lncRNAs are involved in the development of specialized neural tissues such as the retina of the eye. For example, in mouse, the lncRNA RNCR2 plays a critical role in regulating mammalian retinal cell fate specification (Rapicavoli et al., 2010), and the intergenic lncRNA TUG1 appears to favor rod cell specification by inhibiting cone-specific gene expression (Young et al., 2005). Mouse CRNDE is known to be expressed during the development of a subset of cells in the neuroblastic layer of the retina. Its expression has a narrow temporal window, and shifts from inner to outer layer during development (Trimarchi et al., 2007). Such precise and transient expression is consistent with a defined regulatory function.

As discussed above, CRNDE is the most highly upregulated lncRNA in gliomas. The origin of gliomas is a subject of debate, but recent evidence suggests they derive from neural precursors/NSCs; stem cells isolated from these tumors were capable of forming gliomas, glial cells, and neurons, which accords with the capabilities of NSCs (Watkins and Sontheimer, 2012). In any case, the biology of glioma cells resembles that of NSC, in that both are multipotent and express similar stem cell markers (Bao et al., 2006). CRNDE’s upregulation in glioma tumors may serve a similar purpose as it does in neural precursors, i.e., to maintain the stemness of the tumor cells. As long as CRNDE expression is maintained, differentiation into glial cells would be impeded. Upregulation of the EZH2 subunit of PRC2 (a complex with which CRNDE RNA has been shown to associate) has been reported in gliomas, especially glioblastomas, and appears necessary for maintenance of glioblastoma stem cell pluripotency and glioma cell proliferation (Suvà et al., 2009; Orzan et al., 2011). Thus, epigenetic modifications likely play a large part in neurogenesis and brain tumorigenesis.

In summary, the highly specific pattern of CRNDE expression in brain tissue, its involvement in maintenance of stemness, and its association with PRC2 and CoREST chromatin-modifying complexes suggest that CRNDE may be involved in lineage specification in the mammalian brain.

### A potential link to neurodegeneration

Non-coding RNAs, including some lncRNAs, are now proving to be important contributors to neurodegenerative processes (Salta and De Strooper, 2012). Of possible relevance to CRNDE is the review by Watkins and Sontheimer (2012) which provides an interesting discussion of the similarities, mainly in the symptoms and end results, between neurodegenerative disease and glial tumorigenesis. Neurodegeneration is a hallmark of gliomas that is attributed to their characteristic infiltrative growth, which kills existing neurons within surrounding brain tissue (Lee et al., 2011; Watkins and Sontheimer, 2012). Neurodegeneration also results from gliomas’ ability to release the neurotransmitter glutamate, and their inability to clear glutamate from the synaptic cleft due to the epigenetic silencing of its receptors. Overall, this causes glutamate excitotoxicity, which in turn results in numerous ramifications associated with both cancer and neurodegenerative pathologies (Watkins and Sontheimer, 2012). As discussed above, CRNDE is markedly elevated in gliomas and is thus associated with a neurodegenerative process, albeit one resulting from cancer.

There is little in the existing data to link the various neurodegenerative diseases with abnormal CRNDE expression. We have examined CRNDE expression in a gene expression dataset focused on Alzheimer’s disease (AD) and representing 1300 brain tissue samples from 1000 people (Gene Logic, Gaithersburg, MD, USA). AD affects not only the cerebral cortex, where (for the most part) CRNDE is not normally expressed, but is characterized in the earliest stages by degeneration of the hippocampus, a structure in or near the CRNDE-expressing region (Figure 3). After correcting for patient age and gender, no statistically significant correlation was found between CRNDE expression and patient status in regard to AD, neuritic amyloid plaques, or hippocampal neurofibrillary tangles.

Several lncRNAs have been implicated in the hereditary neurodegenerative disorder, Huntington’s disease (HD; Johnson, 2012). One study does show a modest positive correlation between CRNDE expression and HD; this is especially noticeable in the caudate nucleus from patients with grade III HD (2.4-fold increase relative to non-HD control tissue; Hodges et al., 2006). Moreover, an Ingenuity Pathway Analysis of our preliminary microarray studies indicates “HD Signaling” as a highly affected canonical pathway upon knockdown of CRNDE in a CRC cell line (unpublished data). HD is characterized by expansion of the (CAG)_n_ repeat motif in the *huntingtin* gene, which yields a mutant protein. Studies have also investigated the PRC2 complex in HD, and there may be a correlation of wild-type and mutant huntingtin protein with PRC2 activity and H3K27 trimethylation (Seong et al., 2010).

The possible association of CRNDE with HD suggests that it may be rewarding to investigate the expression of its transcripts in hereditary neurodegenerative conditions that involve cell types and brain regions in which the gene is expressed.

## Conclusion

Long non-coding RNAs are increasingly implicated in the molecular mechanisms that underpin normal developmental processes, such as cellular multipotency and differentiation, as well as in the dysregulation that characterizes cancer and neurological disorders (reviewed by Niland et al., 2012). *CRNDE* is a gene that expresses multiple splice variants of a lncRNA and displays a very time- and tissue-specific pattern of expression. It was initially identified as a gene whose expression is highly upregulated in CRC, but it also increases in many other solid tumors and in leukemias. Indeed, CRNDE is the most upregulated lncRNA in gliomas and here, as in other cancers, it is associated with a “stemness” signature. The rise in CRNDE levels during neoplasia may be a response to perturbations in upstream signaling pathways, for example the MAP kinase pathway, which among other actions mediates SRF activity. Regulation by Myc may be a key component of CRNDE expression in stem cells and its overexpression in cancers such as gliomas and CRC, where elevated Myc expression and (in the cancers) gene amplification are common. Cell culture studies using bioactive agents (e.g., hormones, growth factors), specific inhibitor compounds, and knock-in or knock down of specific TFs may shed light on the signaling pathways that control CRNDE expression.

Based on precedents with other lncRNAs (Tsai et al., 2010; Niland et al., 2012), we suggest that CRNDE is a multifunctional lncRNA whose different splice isoforms may provide specific functional scaffolds for regulatory complexes, such as the PRC2 and CoREST chromatin-modifying complexes, which they can pilot to target genes. The promoters of these genes are then downregulated by epigenetic modification. In ESC, CRNDE’s function may be to suppress lineage-determining genes. During the differentiation of certain lineages (e.g., ectodermal cell types), CRNDE levels are known to rise further, perhaps in order to suppress genes that specify alternate cell fates. In cancers of tissues where CRNDE is not normally expressed, its aberrant elevation may again help to suppress lineage markers and revert the cells to a less differentiated state.

The importance of CRNDE to a complete organism can be addressed using CRNDE knock-out mice, with the hope that tissue-specific knock-outs will reveal much about CRNDE’s roles in specialized processes such as gametogenesis and brain function. *In vitro*, the examination of different cell lines in which CRNDE has been knocked in or knocked down should continue to be highly informative about its molecular interactions, with isoform-specific studies helping to distinguish between effects mediated by the exonic and intronic segments of the transcripts. Phenotypic assays on these cells will illuminate the functional consequences of adding or removing individual CRNDE transcripts. Expression microarray surveys will reveal the scope of CRNDE’s target gene set and how this varies between cell types, as well as the extent to which differentiation/lineage markers are included. Established methods such as FISH and new high-resolution methods, including ChIRP (Chu et al., 2011) and CHART (Simon et al., 2011), offer opportunities to determine the full complement of genomic targets bound by CRNDE transcripts. Furthermore, related capture technologies will help to identify proteins not yet known to bind to CRNDE transcripts.

## Conflict of Interest Statement

The authors declare that the research was conducted in the absence of any commercial or financial relationships that could be construed as a potential conflict of interest.

## Footnotes

^1^http://www.nextbio.com

^2^http://www.biogps.org

^3^http://www.genome.ucsc.edu

^4^http://www.cbse.ucsc.edu/~jsp/EvoFold

^5^http://www.ncbi.nlm.nih.gov/geo/query/acc.cgi?acc=GSE26193

^6^http://www.ncbi.nlm.nih.gov/IEB/Research/Acembly/av.cgi?db=human&term=crnde&submit=Go

^7^http://www.ncbi.nlm.nih.gov/UniGene/ESTProfileViewer.cgi?uglist=Hs.237396

^8^http://www.ebi.ac.uk/gxa/experiment/E-GEOD-21668/ENSG00000245694

^9^http://www.ncbi.nlm.nih.gov/sites/GDSbrowser?acc=GDS2431

^10^http://www.ncbi.nlm.nih.gov/sites/GDSbrowser?acc=GDS2615

## Appendix

**Table A1:** **Gene/transcript names and microarray probesets for human and mouse CRNDE**.

Attribute	Human		Mouse	
	Details	Context/source	Details	Context/source
**GENE**				
Location/build (RefSeq gene)	chr16:54,952,777-54,963,101 (NR_034105)	NCBI GRCh37	chr8:94,879,820-94,881,675	NCBI m37
	chr16:54952778-54962690 (NR_034106)	UCSC hg19		UCSC mm9
		Ensembl 68.37		Ensembl v47
Strand	Minus		Minus	
Gene symbol	CRNDE		4933436C20Rik	
Ensembl gene	ENSG00000245694		ENSMUSG00000031736	
UniGene	Hs.237396		Mm.86664	
Vega gene	CRNDE		RP24-69M8.2	
H-InvDB 8.0 ID	HIX0038716	(Cluster ID)		
Locus tag	hCG_1815491	
Gene ID	LOC643911 + LOC388279		LOC626493	
Other gene ID	PNAS-108	AF275804 in GenBank	71296	Entrez GeneID
lncipedia gene	lnc-IRX3-4	lncipedia.org		
**lncRNA**				
	lincIRX5	Khalil et al. (2009)	linc1399	Clark et al. (2012)
	XLOC_011950	Cabili et al. (2011)	linc1399	Guttman et al. (2011)
	(Ensembl gene)	Derrien et al. (2012)	NR_033641	NCBI RefSeq
	NR_034105	NCBI RefSeq (Graham et al., 2011)		
	NR_034106			
	LINC00180		
	NCRNA00180	
**MICROARRAY PROBESETS**				
Affymetrix GeneChip probeset IDs	238021_s_at	Human Genome U133	1431297_a_at	Mouse Genome 430 2.0
	238022_at			
	3692517-3692527	Human Exon 1.0 ST	27 non-consecutive^a^	Mouse Exon 1.0
	3692529, -31, -33			
	3661555, -56, -58	
Agilent SurePrint G3 probeset IDs	A_21_P0008884	Human Gene Expression 8 × 60 K v2	A_51_P378381	Mouse Gene Expression 8 × 60 K
	A_19_P00322533			
	A_32_P104063	
	A_19_P00322531	
	A_19_P00318645	
Illumina expression BeadChips	ILMN_1671800	HumanWG-6 3.0	ILMN_2604403	Sentrix Mouse 6.2

*^a^The 11 probesets that map to RefSeq exons are numbers 4333223, 4615448, 4907780, 4956596, 5051610, 5077062, 5117873, 5219245, 5259826, 5442877, and 5461263*.
